# Improvement in Nighttime Awakening Symptoms After Bracing, Injections, and Carpal Tunnel Release

**DOI:** 10.7759/cureus.94643

**Published:** 2025-10-15

**Authors:** Gopal R Lalchandani, Dafang Zhang, Brandon Earp, Philip Blazar

**Affiliations:** 1 Orthopaedic Surgery, University of California, San Francisco, San Francisco, USA; 2 Orthopedic Surgery, Brigham and Women’s Hospital, Harvard Medical School, Boston, USA; 3 Orthopedics, Harvard Medical School, Boston, USA

**Keywords:** carpal tunnel release, carpal tunnel syndrome, injection, insomnia, nighttime awakening

## Abstract

Background

Carpal tunnel syndrome (CTS) is the most common peripheral compressive neuropathy, and nighttime awakening (NTA) symptoms commonly impact patients with CTS. While carpal tunnel release (CTR) is known to improve NTA, the effect of bracing and corticosteroid injections on NTA remains understudied. We hypothesized that injections and CTR would yield greater early Insomnia Severity Index (ISI) score improvements than bracing.

Methodology

We conducted a prospective, observational, cohort study on patients with CTS treated with bracing (n = 6, 17%), injections (n = 8, 22%), or mini-open CTR (n = 22, 61%) at a single institution between January 2022 and April 2023. All patients received an online questionnaire before treatment and approximately two weeks post-treatment. Questionnaires included the ISI and the Boston Carpal Tunnel Syndrome Questionnaire, including the symptom severity scale and functional status scale.

Results

A total of 36 patients were enrolled, including 11 men (mean age = 65.4) and 25 women (mean age = 56.5). At baseline, the cohort included 78% overweight/obese patients and 6% (n = 2) with diabetes. After a mean of 19.6 days of follow-up, all groups demonstrated improvements in ISI scores (bracing group = 14.7 to 12.7, p = 0.218, injection group = 21.5 to 13.6, p = 0.001, and surgery group = 16.5 to 13.2, p = 0.017). The injection and CTR groups demonstrated significant improvements in univariate analysis. On a multivariable analysis, there was no significant difference between bracing, injections, and CTR in the overall change in symptom scores from baseline.

Conclusions

In this single-institution, prospective study, patients treated with bracing, injections, and surgery for CTS experienced early improvements in NTA and CTS symptoms. On multivariable analysis, neither intervention performed significantly better than the other groups when controlling for comorbidities; however, the analysis was limited by small cohorts, especially in the non-surgical groups.

## Introduction

Carpal tunnel syndrome (CTS) is the most common compressive peripheral neuropathy and is estimated to occur in 3.8% of the general population [1]. First-line treatment for CTS includes nocturnal wrist splinting, but local corticosteroid injections into the carpal tunnel have also been proven effective in many cases [1]. In addition, carpal tunnel release (CTR) is a widely effective surgical treatment for idiopathic CTS [2]. As such, approximately 500,000 CTRs are performed in the United States each year [3]. While early improvement in nighttime awakening (NTA) symptoms after carpal tunnel surgery is a well-known phenomenon [4,5] that is associated with improved patient outcomes [6], improvement in NTA with nocturnal splinting or corticosteroid injections is unknown.

NTA is frequently reported in patients with CTS, although this specific symptom is generally given limited attention [7]. To date, there are limited studies comparing the resolution of NTA after non-operative treatment of CTS. Ly-Pen et al. compared the effects of local steroid injection versus CTR on improvement in nocturnal paresthesias [8]. The investigators found a similar number of wrists that reached greater than 20% improvement in visual analog scores for nocturnal paresthesias across both groups at one year, but they did not evaluate the impact of bracing [8]. Unlike visual analog scores for nocturnal paresthesias, the Insomnia Severity Index (ISI) is an accepted, validated outcome measure used for patients suffering from nocturnal awakening [9] and has been utilized to study outcomes of CTR surgery [4,5].

In this study, we sought to compare patients treated with bracing, corticosteroid injections, and surgery for CTR in terms of their improvement in ISI scores at two weeks after initiation of treatment, as prior studies suggest early improvement in sleep within two weeks after CTR [10]. We hypothesized there would be improvements in patients treated with injections or surgery compared to those treated with bracing in the primary outcome of ISI scores greater than the minimal clinical threshold of 2.5 points [11]. We also sought to evaluate for improvements in the validated Boston Carpal Tunnel Questionnaire (BCTQ) [12] scores as a secondary outcome. These data could prove useful for patient counseling on NTA when seeking treatment for CTS, whether non-operative (wrist bracing or injections) or operative CTR.

## Materials and methods

This pragmatic pilot study prospectively enrolled adult patients at a single institution who received treatment for CTS. Adult patients with CTS over the age of 18 years were included if they were literate and fluent in English. The diagnosis of CTS was confirmed with either electrodiagnostic studies, ultrasound, or the validated Carpal Tunnel Syndrome-6 evaluation tool. Patients were excluded if they had a concomitant upper extremity compressive neuropathy (e.g., cubital tunnel syndrome) or had a recent hand/upper extremity surgery within three months. Patients who did not have a valid email address or access to a computer/smartphone were also excluded, given that this was the mechanism for follow-up surveys. Patients receiving treatment with either nocturnal bracing, injections, or mini-open CTR after a shared decision regarding treatment options was reached between March 2022 and April 2023 were included. Patients treated with bracing were advised to utilize a wrist orthosis to maintain the wrist in a neutral position at night. Patients treated with an injection were treated with a landmark-guided carpal tunnel injection of a combination of a corticosteroid and local anesthetic as per the treating surgeon’s standard practice. Patients undergoing surgery were treated with a mini-open CTR by one of the three surgeons at the same institution.

After obtaining signed electronic consent from each included patient, all patients received a questionnaire including demographic information (name, age, gender, email, laterality, self-reported history of diabetes, the five-item validated ISI, and the 19-item validated BCTQ). Patients filled out this questionnaire on their personal electronic device at the beginning of treatment, during the clinic visit for bracing and injection groups, or in the preoperative area for surgical patients. All patients were emailed the same questionnaire two weeks after the index survey, and electronic reminders were provided within two days.

A subsequent chart review was performed to obtain covariates for later data analysis. Information, including employment, body mass index (BMI), diabetes, hypothyroidism, rheumatoid arthritis, pregnancy, menopause, hemodialysis, renal insufficiency, and congestive heart failure, was ultimately recorded and utilized as covariates in a multivariable analysis.

All statistical analyses were performed using Microsoft Excel with the Analysis Tool Pack. A paired two-sample Student’s t-test was performed to evaluate for differences in pretreatment and post-treatment ISI scores in each treatment group. This was repeated for pre- and post-treatment BCTQ scores. The change in ISI and BCTQ scores was then compared across groups using an analysis of variance (ANOVA) test. Finally, multivariable regression analyses were used to evaluate changes in ISI and BCTQ scores in each treatment group after controlling for employment status and comorbid conditions. Significance was set at 0.05. This study was approved by our Institutional Review Board (approval number: 2010P002462).

## Results

A total of 36 patients were included in the study, including 11 men (mean age = 65.4 years ± 9.6) and 25 women (mean age = 56.5 years ± 17.2). At baseline, the cohort included 78% overweight/obese patients (mean BMI = 30.4 kg/m^2^ + 6.5) and two (6%) patients with diabetes. The average time of follow-up questionnaire completion was 19.6 days. There were six patients in the bracing group, eight patients in the injection group, and 22 patients in the mini-open CTR group who completed the study. Baseline characteristics of each treatment group are described in Table 1.

**Table 1:** Baseline characteristics of all treatment groups.

Patient characteristics	Brace (n = 6)	Injection (n = 8)	Surgery (n = 22)
Average age (years)	62.83	51.50	56.68
Average body mass index (kg/m²)	29.40	31.28	30.29
Gender: number of males (%)	3 (50%)	1 (13%)	7 (32%)
Employment: number of employed individuals (%)	4 (67%)	6 (75%)	15 (68%)
Diabetes mellitus	1 (17%)	1 (13%)	2 (9%)
Hypothyroidism	1 (17%)	1 (13%)	3 (14%)
Rheumatoid arthritis	0 (0%)	0 (0%)	1 (5%)
Pregnancy	0 (0%)	0 (0%)	1 (5%)
Menopause	0 (0%)	0 (0%)	4 (18%)
Renal insufficiency	0 (0%)	0 (0%)	3 (14%)
Congestive heart failure	0 (0%)	0 (0%)	1 (5%)
Smoking: number of former/current smokers (%)	3 (50%)	3 (37.5%)	10 (45%)

After a mean of 19.6 days of follow-up, all three groups demonstrated improvements in their ISI scores, as well as BCTQ scores. Mean preoperative ISI scores in the bracing group were 14.7, and postoperative ISI scores were 12.7, a trend toward improvement that was not statistically significant (p = 0.218). However, improvements in the injection group (21.5 to 13.6, p = 0.001) and the surgery group (16.5 to 13.2, p = 0.017) were statistically significant (Table 2). We found similar improvements in both the Symptom Severity Score and Functional Severity Score of the BCTQ, which were not statistically significant in the bracing group but significantly improved in the injection and surgery group (Tables 3, 4).

**Table 2:** Comparison of preoperative and postoperative ISI scores in each treatment group with a paired t-test. ISI = Insomnia Severity Index

Treatment group	Preoperative ISI, mean	Postoperative ISI, mean	P-value
Brace (n = 6)	14.67	12.67	0.218
Injection (n = 8)	21.50	13.63	0.001
Surgery (n = 22)	16.55	13.23	0.017

**Table 3:** Comparison of preoperative and postoperative SSS scores in each treatment group with a paired t-test. SSS = Symptom Severity Scale

Treatment group	Preoperative SSS, mean	Postoperative SSS, mean	P-value
Brace (n = 6)	2.52	2.26	0.289
Injection (n = 8)	2.85	1.86	0.001
Surgery (n = 22)	2.98	1.88	<0.001

**Table 4:** Comparison of preoperative and postoperative FSS scores in each treatment group with a paired t-test. FSS = Functional Severity Scale

Treatment group	Preoperative FSS, mean	Postoperative FSS, mean	P-value
Brace (n = 6)	2.27	1.65	0.162
Injection (n = 8)	1.72	1.31	0.007
Surgery (n = 22)	2.31	1.74	0.002

When comparing the improvement across all three groups, there was no statistically significant improvement in ISI, SSS, or FSS scores. Table 5 demonstrates ANOVA results comparing all three outcomes across all three groups. While the largest numeric magnitude of improvement in ISI scores occurred in the injection group, this was not significantly different when compared to the other two treatment groups.

**Table 5:** Comparison of change in ISI, SSS, and FSS scores across all three treatment groups with ANOVA. ISI = Insomnia Severity Index; SSS = Symptom Severity Scale; FSS = Functional Severity Scale; ANOVA = analysis of variance

Treatment group	Change in ISI	Change in SSS	Change in FSS
Brace (n = 6)	2.00	0.26	0.63
Injection (n = 8)	7.88	0.99	0.41
Surgery (n = 22)	3.32	1.10	0.57
P-value	0.165	0.131	0.870

When controlling for age/gender, employment status, and multiple comorbidities, we again did not identify a statistically significant difference in ISI, SSS, or FSS scores between treatment groups (Tables 6-8). Comorbidities included in our multivariable analysis were obesity, diabetes, hypothyroidism, rheumatoid arthritis, pregnancy, menopause, hemodialysis, renal insufficiency, and congestive heart failure.

**Table 6:** Multivariable regression analysis of change in ISI across all three treatment groups. ISI = Insomnia Severity Index

Change in ISI	Standard error	P-value	Upper 95%	Lower 95%
Brace (n = 6)	Reference	Reference	Reference	Reference
Injection (n = 8)	3.87	0.312	12.07	-4.04
Surgery (n = 22)	3.43	0.506	4.81	-9.45
Age	0.18	0.302	0.18	-0.56
Gender	3.49	0.894	7.72	-6.78
Employment	5.03	0.909	9.89	-11.04
Body mass index (kg/m²)	0.21	0.614	0.34	-0.56
Diabetes mellitus	4.13	0.434	11.88	-5.29
Hypothyroidism	3.80	0.342	4.21	-11.60
Rheumatoid arthritis	9.99	0.677	25.00	-16.56
Pregnancy	7.44	0.511	20.44	-10.49
Menopause	4.00	0.289	12.68	-3.97
Renal insufficiency	5.76	0.096	22.03	-1.94
Congestive heart failure	8.29	0.538	22.44	-12.05
Smoking (current or prior)	0.78	0.856	1.77	-1.49

**Table 7:** Multivariable regression analysis of change in SSS across all three treatment groups. SSS = Symptom Severity Scale

Change in SSS	Standard error	P-value	Upper 95%	Lower 95%
Brace (n = 6)	Reference	Reference	Reference	Reference
Injection (n = 8)	0.45	0.439	1.29	-0.58
Surgery (n = 22)	0.40	0.356	1.21	-0.45
Age	0.02	0.092	0.01	-0.08
Gender	0.41	0.467	0.54	-1.15
Employment	0.59	0.520	0.84	-1.60
Body mass index (kg/m²)	0.03	0.204	0.02	-0.08
Diabetes mellitus	0.48	0.400	1.41	-0.59
Hypothyroidism	0.44	0.143	0.25	-1.59
Rheumatoid arthritis	1.16	0.070	4.65	-0.19
Pregnancy	0.87	0.728	1.50	-2.11
Menopause	0.47	0.306	1.46	-0.48
Renal insufficiency	0.67	0.879	1.50	-1.29
Congestive heart failure	0.97	0.462	2.73	-1.29
Smoking (current or prior)	0.09	0.453	0.12	-0.26

**Table 8:** Multivariable regression analysis of change in FSS across all three treatment groups. FSS = Functional Severity Scale

Change in FSS	Standard error	P-value	Upper 95%	Lower 95%
Brace (n = 6)	Reference	Reference	Reference	Reference
Injection (n = 8)	0.49	0.383	0.58	-1.46
Surgery (n = 22)	0.43	0.749	0.76	-1.04
Age	0.02	0.160	0.01	-0.08
Gender	0.44	0.990	0.91	-0.92
Employment	0.64	0.991	1.32	-1.33
Body mass index (kg/m²)	0.03	0.356	0.03	-0.08
Diabetes mellitus	0.52	0.252	1.70	-0.47
Hypothyroidism	0.48	0.977	0.99	-1.02
Rheumatoid arthritis	1.27	0.710	3.11	-2.16
Pregnancy	0.94	0.896	1.83	-2.09
Menopause	0.51	0.695	1.26	-0.85
Renal insufficiency	0.73	0.333	0.80	-2.24
Congestive heart failure	1.05	0.977	2.16	-2.22
Smoking (current or prior)	0.10	0.424	0.13	-0.29

## Discussion

In this prospective review of 36 patients treated for CTS at a single institution, patients treated with injections and surgery demonstrated significantly improved ISI and BCTQ scores compared to pretreatment levels at an average of 19.6 days after treatment. The mean ISI improvement after injections was a mean of 7.9, and the mean improvement after CTR was 3.3, which is well above the minimal clinically important difference in ISI scores, which has been reported at 2.4 to 2.6 [11].

While patients did demonstrate significant improvements compared to pretreatment scores in the injection and surgery groups, our subsequent analyses did not reveal a significant difference when comparing all three treatment groups, even when controlling for multiple comorbidities. This may be due to the small number of patients in each group. Particularly in patients treated non-surgically, this study enrolled six patients treated with bracing and eight patients treated with injection, which is certainly a limitation of this study.

When comparing the results of this study to historical data, the early improvement in NTA noted in this study is consistent with prior studies of CTR. Niedermeier et al. studied NTA after CTR, and they reported rapid and sustained improvement within two weeks of CTR [10]. In addition, Watchmaker et al. retrospectively reviewed 1,131 consecutive patients undergoing open CTR and found consistent improvements in nocturnal awakening across all age groups [13]. Our results are consistent with these findings, but also support improvements in NTA after corticosteroid injections over a similar time period. In fact, the level of improvement in ISI scores was almost double after an injection when compared to CTR, but this difference was not statistically significant between groups, potentially due to small sample sizes. These findings are consistent with multiple prior studies that support similar short-term symptomatic improvement with both injections and CTR [8,14]. This study is strengthened by its prospective inclusion of bracing as a comparison cohort, which prior studies have not included.

While some may interpret these results to potentially support more routine use of carpal tunnel injections in the treatment of CTS, it is important to recognize that this study only looked at improvement in patient-reported scores and did not quantify improvements in objective clinical examination findings, electrodiagnostic, or sonographic parameters. In fact, Andreu et al. conducted a randomized controlled trial comparing injections and CTR, reporting that only decompressive surgery allows resolution of neurophysiologic changes [15]. Regardless, these results can be useful for patient counseling when discussing the short-term impact of injections on NTA, as this has not yet been well described. However, the durability of this effect is unknown.

Although the results of this study will be helpful with patient guidance in the treatment of NTA in CTS, there are certainly limitations with these data. As suggested previously, the limited numbers in the bracing and injection groups limit the strength of our comparisons between treatment groups. In addition, the lack of treatment randomization makes it difficult to control for selection bias and confounding factors, such as disease severity, that may preferentially impact one treatment group selectively. We sought to mitigate this concern by controlling for multiple medical comorbidities in our multivariable analysis, which ultimately did not demonstrate a difference between treatment groups. Finally, while the reporting of prospectively collected patient-reported data is a strength of this study, the lack of objective electrodiagnostic, sonographic, or clinical examination findings after treatment is certainly a limitation. The lack of these data makes further comparisons of the three treatment groups more difficult.

Despite these shortcomings, this study does prompt areas for future studies in the treatment of CTS. In this small cohort, both CTR and corticosteroid injection demonstrated clinically meaningful improvements in nocturnal awakening symptoms in the short-term, but larger randomized trials are necessary to confirm the durability of these findings in the mid- and long-term. In addition, the use of electrodiagnostic and sonographic data in future studies can help understand structural changes after each intervention and compare improvement over time.

## Conclusions

In this single-institution, prospective study, patients treated with injections and CTR for CTS experienced early significant improvements in NTA symptoms well above the minimally clinically important difference. These results can be helpful when counseling patients, as neither intervention performed significantly better than the other treatments when controlling for comorbidities. Future research in the field is necessary to help further clarify optimal treatments for patients troubled by NTA due to CTS.
